# *GSTT2 *promoter polymorphisms and colorectal cancer risk

**DOI:** 10.1186/1471-2407-7-16

**Published:** 2007-01-25

**Authors:** Sang-Geun Jang, Il-Jin Kim, Hio Chung Kang, Hye-Won Park, Sun-A Ahn, Hyun-Ju Yoon, Kun Kim, Hai-Rim Shin, Jin Soo Lee, Jae-Gahb Park

**Affiliations:** 1 Laboratory of Cell Biology, Cancer Research Institute and Cancer Research Center, Seoul National University, Seoul, Korea; 2 Research Institute and Hospital, National Cancer Center, Goyang, Gyeonggi, Korea

## Abstract

**Background:**

Glutathione *S*-transferases are a group of enzymes that participate in detoxification and defense mechanisms against toxic carcinogens and other compounds. These enzymes play an important role in human carcinogenesis. In the present study, we sought to determine whether *GSTT2 *promoter single nucleotide polymorphisms (SNPs) are associated with colorectal cancer risk.

**Methods:**

A total of 436 colorectal cancer patients and 568 healthy controls were genotyped for three *GSTT2 *promoter SNPs (-537G>A, -277T>C and -158G>A), using real-time TaqMan assay and direct sequencing. An electrophoretic mobility shift assay (EMSA) was performed to determine the effects of polymorphisms on protein binding to the *GSTT2 *promoter.

**Results:**

The -537A allele (-537G/A or A/A) was significantly associated with colorectal cancer risk (OR = 1.373, *p *= 0.025), while the -158A allele (-158G/A or A/A) was involved in protection against colorectal cancer (OR = 0.539, *p *= 0.032). Haplotype 2 (-537A, -277T, -158G) was significantly associated with colorectal cancer risk (OR = 1.386, *p *= 0.021), while haplotype 4 (-537G, -277C, -158A) protected against colorectal cancer (OR = 0.539, *p *= 0.032). EMSA data revealed lower promoter binding activity in the -537A allele than its -537G counterpart.

**Conclusion:**

Our results collectively suggest that SNPs and haplotypes of the *GSTT2 *promoter region are associated with colorectal cancer risk in the Korean population.

## Background

Colorectal cancer (CRC), a predominant cause of tumor-related death in Western nations, is becoming more prevalent in Asian countries such as Korea [1,2]. Genetic and environmental factors are modulators of the carcinogenesis process [3]. Studies on the contribution of genetic polymorphisms to cancer development have examined numerous genes, including oncogenes, tumor suppressors, DNA repair genes and those encoding Phase I and Phase II enzymes [4]. Phase II enzymes attach to additional substrates in an attempt to detoxify the activated metabolite in preparation for final breakdown or excretion. Glutathione-*S*-transferases (GSTs) are a family of Phase II detoxification enzymes that protect cellular macromolecules by catalyzing the conjugation of glutathione (GSH) to a wide variety of endogenous and exogenous electrophilic compounds [5]. GSTs are divided into at least six classes (alpha, mu, pi, theta, omega and zeta), each of which consists of one or more isoforms [6]. GST polymorphisms are associated with bladder, colorectal, gastric, prostate and various other human cancers [5,7-11].

The theta class consists of two genes, *GSTT1 *and *GSTT2*, located at 22q11.2, and separated by about 50 kb. *GSTT1 *and *GSTT2 *share 55% amino acid sequence identity [12]. The *GSTT1*-null genotype is associated with increased CRC risk [7,13,14]. A common characteristic of the theta class is their affinity for the organic hydroperoxide species; for example, cumene hydroperoxide is a good substrate for GSTT2 [15]. Thus, GSTT2 activity is important for the protection of cells against toxic products of oxygen and lipid peroxidation [16], which represents a major source of endogenous DNA damage in humans that contributes significantly to cancer and other genetic diseases [17]. Pool-Zobel *et al*. [18] observed that *GSTT2 *involved in defense against oxidative stress in primary human colon cells is up-regulated upon incubation with butyrate. A comprehensive study of Phase I and Phase II metabolism gene polymorphisms revealed no significant contribution of *GSTT2 *polymorphism to CRC risk [19]. However, distinct effects of *GSST2 *promoter haplotypes on gene expression were observed with a luciferase reporter assay [20]. Genetic alterations may induce abnormal gene expression. To date, no case-control analysis of *GSTT2 *promoter polymorphisms has been performed in human cancers.

In the present study, we sought to determine whether four *GSTT2 *promoter polymorphisms, -537G>A, -277T>C, -158G>A, and -129T>C, and their haplotypes are associated with CRC risk. We additionally employed EMSA to determine whether *GSTT2 *promoter polymorphisms affect the binding of putative transcription factors.

## Methods

### Samples and DNA extraction

Four hundred and thirty-six colorectal cancer samples were collected from the Seoul National University Hospital and the National Cancer Center Hospital, Korea. Clinical characteristics, including age, sex, tumor location, and TNM stage, were additionally analyzed for the association study with *GSTT2 *genotyping. DNA was extracted from normal colorectal tissues. Fresh cancer tissues were stored at -70°C in a liquid nitrogen tank. Normal population samples were obtained from 568 healthy Korean individuals as controls, and DNA was isolated from blood samples. All colorectal cancer samples were collected from Korean patients enrolled between 1990 and 2003 at Seoul National University and National Cancer Center. Normal controls were selected from cancer-free samples enrolled from the Cancer Cohort Study Branch of the National Cancer Center. The mean ages of colorectal cancer patients at diagnosis and normal subjects were 58.8 years (58.8 ± 12.9 years) and 52.1 years (52.1 ± 10.5 years), respectively.

Total genomic DNA was extracted with TRIzol reagent, according to the manufacturer's instructions (Invitrogen, CA, USA). This study was approved by the institutional review board of the National Cancer Center and informed consent was obtained from all subjects prior to inclusion in the study.

### GSTT2 genotyping

The -537G>A (rs140186) and -277T>C (rs9624369) polymorphisms were screened using real-time TaqMan assay, and -158G>A and -129T>C polymorphisms were screened by direct sequencing. For the TaqMan assay, both PCR primers and MGB TaqMan probes were designed using the commercial Assay by Design service (Applied Biosystems, Foster City, CA, USA). One allelic probe was labeled with FAM dye, and the other with VIC. PCR was performed using the TaqMan Universal Master MIX without UNG in a 7900 HT fast real-time system (Applied Biosystems, Foster City, CA, USA). Data files were assessed using SDS2.1.1 software (Applied Biosystems, Foster City, CA, USA). We used BigDye Terminator and an ABI PRISM 3730 DNA analyzer (Applied Biosystems, Foster City, CA, USA) for direct sequencing analysis. Amplification for direct sequencing was performed in a final volume of 15 μl containing 10 ng genomic DNA, 10 pmol of each primer (forward: 5'-CCACTGGGTGAAACTCTAAG-3', reverse: 5'-GGACACCAGGTCAAGAAAC-3'), 0.25 mM each dNTP, 0.5 U of *Taq *polymerase and reaction buffer (Genecraft Ltd, Munster, Germany). Reactions were performed in a programmable thermal cycler (MWG Biotech AG, Eberberg, Germany) under the following conditions: denaturation for 5 min at 94°C, followed by 40 cycles at 94°C for 30 s, 55°C for 30 s, 72°C for 1 min, and a final extension of 10 min at 72°C.

### Microsatellite instability (MSI) analysis

Two microsatellite markers (BAT-25 and BAT-26) were used to assess MSI status. DHPLC (denaturing high-performance liquid chromatography) and the capillary-based method were employed [2]. Cancer samples were classified as MSI when at least one MSI was evident in two markers. MSI data were obtained from 200 previously reported samples [2] and 236 patients analyzed in this study.

### Electrophoretic mobility shift assay (EMSA)

EMSA was performed with the Gel Shift Assay System (Promega Corporation, Madison, WI, USA). Briefly, the following consensus oligonucleotide pairs corresponding to the *GSTT2 *promoter sequence were synthesized (Bioneer, Seoul, South Korea) (bold letters specify polymorphisms): -537G (including the G allele) forward 5'-TAAGATCCCTTTTAG**G**GGATCCCATTCGCTC-3' and reverse 5'-GAGCGAATGGGATCC**C**CTAAAAGGGATCTTA-3', -537A (including the A allele) forward 5'-TAAGATCCCTTTTAG**A**GGATCCCATTCGCTC-3', and reverse 5'-GAGCGAATGGGATCC**T**CTAAAAGGGATCTTA-3'; -158G (including the G allele) forward 5'-AGGAACCGAAGGGGC**G**AGGCGGGTCCGGGGG-3' and reverse 5'-TCCTTGGCTTCCCCG**C**TCCGCCCAGGCCCCC-3', -158A (including the A allele) forward 5'-AGGAACCGAAGGGGC**A**AGGCGGGTCCGGGGG-3', and reverse 5'-TCCTTGGTTCCCCG**T**TCCGCCCAGGCCCCC-3'. Primer pairs were annealed and labeled with [γ-^32^P]ATP (Amersham Biosciences, Buckinghamshire, UK). Binding reactions were performed using HeLa nuclear extracts (Promega Corporation, Madison, WI, USA), according to the manufacturer's instructions. The ^32^P-labeled probe (1 μl) was incubated with 5 mg of HeLa nuclear extracts for 1 h at room temperature. Following the binding reaction, DNA-protein complexes were resolved by electrophoresis on a 4% non-denaturing acrylamide gel, which was subsequently transferred to 3 M blotting paper, dried, and exposed to film.

### Statistical analysis

*GSTT2 *genotyping results were analyzed for categorical variables using the χ2 test, and for continuous variables using student's *t*-test (SPSS 12.0, Chicago, IL). We examined Lewontin's *D' *(|*D'*|) and the linkage disequilibrium coefficient, *r*^2^, between all pairs of biallelic loci [21,22]. Haplotypes of each individual were inferred with the algorithm developed by Stephens *et al*. [21,22], which uses a Bayesian approach incorporating prior expectations of haplotypic structure based on population genetics and the coalescent theory (PHASE version 2.0). The genotype and haplotype-specific risks were estimated as odds ratios (OR) with associated 95% confidence intervals (CI) by logistic regression (SPSS 12.0, Chicago, IL). The observed genotype frequencies were analyzed with a chi-square test to determine whether they were in Hardy-Weinberg equilibrium (HWE). A *p *value of less than 0.05 was considered statistically significant.

## Results and discussion

We initially selected three single nucleotide polymorphisms (SNPs) within the *GSTT2 *promoter region (-537G>A, -277T>C and -129T>C) highlighted in an earlier study [20] and further identified a novel -158G>A polymorphism. The rare -129T>C polymorphism with less than 1% minor allele frequency (MAF) was excluded, and three *GSTT2 *promoter polymorphisms (-537G>A, -277T>C and -158G>A) were analyzed for CRC risk. The MAF values of the three SNPs were 0.42 (-537A), 0.07 (-277C) and 0.03 (-158A) (Table 1). In control samples, the genotype distribution did not deviate from HWE (Table 1). The *GSTT2 *genotype results were not influenced by other potential CRC risk factors, such as age, sex, location, and TNM stage (Table 2). Moreover, the MSI status was not associated with *GSTT2 *polymorphisms (Table 2). Genotypes were analyzed for association with CRC risk using logistic regression models adjusted for age and sex (Table 1). The frequency of the -537A allele (G/A or A/A) was significantly increased in CRC patients compared to healthy controls (OR = 1.373, *p *= 0.025, Table 1). In contrast, the -158A allele (G/A or A/A) was more frequently observed in healthy controls than CRC patients (OR = 0.539, *p *= 0.032, Table 1). Moreover, the -277T/C polymorphism was not related to CRC risk (*p *= 0.125).

Linkage disequilibrium (LD) was tested for all SNP pairs. Lewontin's *D' *values of the pairs were 0.965 (-537G>A:-277T>C), 1.000 (-537G>A:-158G>A) and 1.000 (-277T>C:-158G>A), and the *r*^2 ^values were 0.043, 0.017, and 0.385, respectively. Good level of LD (*D' *> 0.965) was observed between all SNP pairs. Four haplotypes with more than 2% frequency were selected for haplotype association analysis. Haplotype frequencies were 0.511 (HT1), 0.422 (HT2), 0.033 (HT3), and 0.032 (HT4) (Table 3). Haplotypes were analyzed for association with CRC risk using logistic regression models adjusted for age and sex (Table 3). Haplotype 2 (HT2; -537A, -277T, -158G) was correlated with significantly increased CRC risk, compared to non-HT2 (OR = 1.386, *p *= 0.021, Table 3). In contrast, haplotype 4 (HT4; -537G, -277C, -158A) was associated with protection against CRC, compared with non-HT4 (OR = 0.539, *p *= 0.032, Table 3). No significant relationship with CRC risk was evident for haplotypes 1 (HT1; -537G, -277T, -158G) (*p *= 0.336) and 3 (HT3; -537G, -277C, -158G) (*p *= 0.984). The -537A polymorphism positively associated with CRC risk was only observed in HT2, while the -158A polymorphism with a protective role against cancer was present in HT4. Individual SNP analysis of -537G>A and -158G>A is almost consistent with HT2 and HT4, respectively. This may be due to limited individual SNP numbers.

After Bonferroni correction (the threshold of significance was 0.017, 3 polymorphisms were analyzed), the associated *p *values did not retain significance. However, since SNPs within the same gene displayed good level of LD, test statistics for the 3 polymorphisms were not independent, and the significance of association with CRC risk is noteworthy. However, further functional evidence is required to confirm our results that *GSTT2 *promoter polymorphisms are associated with CRC risk.

**Figure 1:**
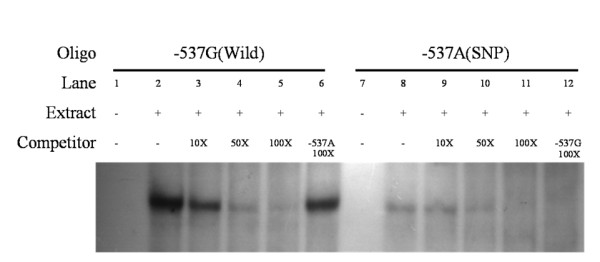
**EMSA with HeLa nuclear extracts using -537G and -537A oligonucleotides**. Binding activities of [γ-^32^P] ATP-labeled -537G (lane 1–6) and -537A (lane 7–12) oligonucleotides. The assay was performed in the presence (+) or absence (-) of HeLa nuclear extracts. Unlabeled -537G or -537A oligonucleotides were used in competition assays. Each binding reaction contained 5 mg of HeLa nuclear extracts and labeled -537G (lanes 2–6) or -537A (lanes 8–12) oligonucleotides. Excess unlabeled oligonucleotides (10-, 50- and 100-fold) were included in the binding reactions as competitors (Lanes 3–5 and 9–11, respectively). In addition, we added a 100-fold excess of unlabeled -537A and -537G oligonucleotides to compete with -537G (Lane 6) and -537A (Lane 12) oligonucleotides. The binding activity of -537G was unaffected, even in the presence of a 100-fold excess of -537A competitor (lane 6). However, the -537A oligonucleotide could not bind transcription factor (lane 12), and displayed no band in the presence of a 100-fold excess of -537G probe.

Accordingly, to determine whether the two polymorphisms affect the binding of putative transcription factors, EMSA was performed using allele-specific consensus oligonucleotide probes. Upon incubation of radiolabeled oligonucleotides specific for -537G and -537A in the presence of HeLa nuclear extracts, the former oligonucleotide displayed stronger band intensity than the latter (Lanes 2 and 8) (Figure 1). To confirm the presence of the DNA-protein complex, competition assays were performed with increasing amounts of unlabeled oligonucleotides (10-, 50-, 100-fold excess). Band densities decreased with increasing concentrations of unlabeled specific competitor (Lanes 3–5 and Lanes 9–11). In the presence of unlabeled non-specific probes, such as a 100-fold excess of the -537A competitor, the -537G oligonucleotide remained unaffected (lane 6). However, the -537A oligonucleotide probe could not bind the transcription factor (lane 12), and displayed no band with a 100-fold excess of -537G probe. Therefore, we conclude that the -537G oligonucleotide has more specific DNA-protein binding capacity than the -537A probe. EMSA was performed to investigate the binding between the -537G and A alleles in HeLa nuclear extracts. The -537A allele displayed weak transcription factor binding activity compared to the -537G allele. The -158G/A polymorphism did not display differences in binding activity in EMSA. Our results strongly indicate that the -537G/A polymorphism of *GSTT2 *specifically influences transcription factor binding activity, leading to a decrease or increase in *GSTT2 *expression.

GSTT2 exhibits high glutathione peroxidase activity with cumene hydroperoxide as a substrate [15]. In human colon cancer cells, butyrate and flavonoids that contribute to detoxification of dietary carcinogens induce upregulation of *GSTT2 *to protect against toxic products of oxygen and lipid peroxidation [18,23]. This gene may have an important role in carcinogenesis and sensitivity of tumors against oxidation stress. *GSTT2 *promoter polymorphisms significantly reduce luciferase activity in two human cell lines (HEK239t and TE671) [18]. SNPs or mutations within the promoter region reportedly affect transcription activity and gene expression [24]. Therefore, SNPs and haplotypes of the *GSTT2 *promoter region result in distinct GSTT2 activities, and the -537A allele may be associated with cancer development arising from oxidation stress.

## Conclusion

In summary, case-control analysis of CRC patients reveals that *GSTT2 *polymorphisms are associated with CRC risk. Individual SNP analyses and haplotype results were consistent. EMSA results revealed lower promoter binding activity of the -537A allele, compared to the -537G allele. To our knowledge, this is the first report that *GSTT2 *promoter polymorphisms and their haplotypes are associated with colorectal cancer risk. Our data collectively suggest that *GSTT2 *promoter polymorphisms are involved in CRC development, and it would thus be beneficial to include *GSTT2 *promoter SNPs when screening for relationships between GST families and human cancers.

## Competing interests

The author(s) declare that they have no competing interests.

## Authors' contributions

SGJ, IJK, and HCK participated in the design of the study, and drafted and wrote the manuscript. SGJ, IJK, HCK, and HWP participated in the statistical analysis. SAA and HJY participated in the production of genotype data. SGJ and KK carried out the EMSA, and HRS and JSL participated in the acquisition and interpretation of data. JGP participated in the design and coordination of the study. All authors read and approved the final manuscript.

## Pre-publication history

The pre-publication history for this paper can be accessed here:



## Figures and Tables

**Table 1:** *GSTT2 *polymorphisms and colorectal cancer risk

**Locus**	**HWE**	**MAF**	**Genotype**	**Healthy controls (N = 568)**	**CRC patients (N = 436)**	***p***	**OR(95% CI)**
-537	0.999	0.42	GG	203 (35.7%)	127 (29.1%)	**0.025**	**1.373 (1.041–1.810)**
			GA or AA	365 (64.3%)	309 (70.9%)		
-277	0.548	0.07	TT	489 (86.1%)	388 (89.0%)	0.125	0.735 (0.495–1.090)
			TC or CC	79 (13.9%)	48 (11.0%)		
-158	0.314	0.03	GG	522 (91.9%)	416 (95.4%)	**0.032**	**0.539 (0.307–0.947)**
			GA or AA	46 (8.1%)	20 (4.6%)		

Adjusted for age and sex; HWE: Hardy-Weinberg equilibrium for controls; MAF: Minor allele frequency

Dominant model: Homozygote of major allele vs. heterozygote and homozygote of minor allele

**Table 2:** Clinical characteristics of colorectal cancer patients and *GSTT2 *genotypes

	**-537 G>A**						**-277T>C**						**-158G>A**		
	
	**GG (%)**	**GA (%)**	**AA (%)**	***p* ^1^**	**GA+AA (%)**	***p* ^2^**	**TT (%)**	**TC (%)**	**CC (%)**	***p* ^1^**	**TC+CC (%)**	***p* ^2^**	**TT (%)**	**TC (%)**	***p* ^2^**
No. of subjects	127	240	69		309		388	45	3		48		416	20	
Age (years ± SD)	59.1 ± 13.2	58.91 ± 13.0	57.9 ± 12.2	0.83	58.7 ± 12.8	0.76	58.6 ± 13.0	60.0 ± 11.7	66.5 ± 2.1	0.56	60.3 ± 11.5	0.41	58.8 ± 13.0	58.2 ± 11.7	0.85
Sex (M/F)															
Male	81 (64.8)	151 (63.4)	44 (63.8)	0.97	195 (63.5)	0.8	247 (64.2)	27 (60.0)	2 (100)	0.49	29 (61.7)	0.74	262 (63.4)	14 (73.7)	0.36
Female	44 (35.2)	87 (36.6)	25 (36.2)		112 (36.5)		138 (35.8)	18 (40.0)	-		18 (38.3)		151 (36.6)	5 (26.3)	
Location															
proxim al	48 (39.3)	84 (35.6)	19 (29.7)	0.43	103 (34.3)	0.33	135 (36.0)	15 (33.3)	1 (50.0)	0.86	16 (34.0)	0.79	141 (35.0)	10 (52.6)	0.18
distal	74 (60.7)	152 (64.4)	45 (70.3)		197 (65.7)		240 (64.0)	30 (66.7)	1 (50.0)		31 (66.0)		262 (65.0)	9 (47.4)	
MSI status															
MSI	17 (13.9)	35 (14.8)	10 (14.5)	0.97	45 (14.8)	0.83	52 (13.6)	10 (23.8)	-	0.17	10 (22.7)	0.1	59 (14.4)	3 (17.6)	0.71
MSS	105 (86.1)	201 (85.2)	59 (85.5)		260 (85.2)		331 (86.4)	32 (76.2)	2 (100)		34 (77.3)		351 (85.6)	14 (82.4)	
T-stage															
T1	-	2 (0.9)	2 (3.1)	0.11	4 (1.3)	0.39	4 (1.1)	-	-	0.95	-	0.74	4 (1.0)	-	0.9
T2	8 (6.5)	8 (3.4)	2 (3.1)		10 (3.3)		15 (4.0)	3 (6.8)	-		3 (6.5)		17 (4.2)	1 (5.3)	
T3	102 (82.3)	195 (83.0)	56 (86.1)		251 (83.4)		317 (83.6)	34 (77.3)	2 (100)		36 (78.3)		336 (82.8)	17 (89.5)	
T4	14 (11.3)	30 (12.8)	5 (7.7)		35 (11.6)		42 (11.1)	7 (15.9)	-		7 (15.2)		48 (11.8)	1 (5.3)	
N-stage															
N0	58 (46.8)	119 (50.4)	35 (53.0)	0.31	154 (51.0)	0.54	189 (49.7)	23 (52.3)	-	0.13	23 (50.0)	0.55	200 (49.1)	12 (63.2)	0.38
N1	36 (29.0)	75 (31.8)	14 (21.2)		89 (29.5)		114 (30.0)	9 (20.5)	2 (100)		11 (23.9)		122 (30.0)	3 (15.8)	
N2	30 (24.2)	42 (17.8)	17 (25.8)		59 (19.5)		77 (20.3)	12 (27.3)	-		12 (26.1)		85 (20.9)	4 (21.1)	
M-stage															
M0	90 (74.4)	182 (78.1)	52 (78.8)	0.69	234 (78.3)	0.39	293 (78.3)	30 (68.2)	1 (50.0)	0.21	31 (67.4)	0.1	311 (77.6)	13 (68.4)	0.35
M1	31 (25.6)	51 (21.9)	14 (21.2)		65 (21.7)		81 (21.7)	14 (31.8)	1 (50.0)		15 (32.6)		90 (22.4)	6 (31.6)	
TNM stage															
I	6 (5.0)	10 (4.3)	3 (4.6)	0.94	13 (4.4)	0.69	16 (4.3)	3 (6.8)	-	0.37	3 (6.5)	0.2	18 (4.5)	1 (5.3)	0.46
II	42 (34.7)	93 (40.1)	28 (43.1)		121 (40.7)		145 (39.0)	18 (40.9)	-		18 (39.1)		154 (38.6)	9 (47.4)	
III	42 (34.7)	78 (33.6)	20 (30.8)		98 (33.0)		130 (34.9)	9 (20.5)	1 (50.0)		10 (21.7)		137 (34.3)	3 (15.8)	
IV	31 (25.6)	51 (22.0)	14 (21.5)		65 (21.9)		81 (21.8)	14 (31.8)	1 (50.0)		15 (32.6)		90 (22.6)	6 (31.6)	

Student's *t*-test or χ^2 ^test; *p*^1^: Co-dominant model; *p*^2^: Dominant model

**Table 3:** Haplotype distribution in colorectal cancer patients and normal controls

**Haplotype**	**Frequecy**	**Distribution**	**Healthy control (N = 568)**	**CRC patients (N = 436)**	***p***	**OR(95% CI)**
HT1 (G-T-G)	0.511	non-HT1/non-HT1	140 (24.6%)	99 (22.7%)	0.336	1.160 (0.857–1.571)
		HT1/non-HT1 or HT1/HT1	428 (75.4%)	337 (77.3%)		
HT2 (A-T-G)	0.422	non-HT2/non-HT2	204 (35.9%)	127 (29.1%)	**0.021**	**1.386 (1.052–1.828)**
		HT2/non-HT2 or HT2/HT2	364 (64.1%)	309 (70.9%)		
HT3 (G-C-G)	0.033	non-HT3/non-HT3	532 (93.7%)	405 (92.9%)	0.984	0.995 (0.595–1.663)
		HT3/non-HT3	36 (6.3%)	31 (7.1)		
HT4 (G-C-A)	0.032	non-HT4/non-HT4	522 (91.9%)	416 (95.4%)	**0.032**	**0.539 (0.307–0.947)**
		HT4/non-HT4	46 (8.1%)	20 (4.6%)		

Adjusted for age and sex; Non-HT1 includes HT2, HT3 and HT4 but not HT1. Dominant model
